# Overview of the sterile insect technique for *Aedes aegypti* in Lee County, Florida, USA

**DOI:** 10.1186/s40249-025-01307-7

**Published:** 2025-05-09

**Authors:** Rachel Morreale, Steven Stenhouse, Johanna Bajonero, Danilo O. Carvalho, Nicole Foley, Roxanne Connelly, Aaron Lloyd, David Hoel

**Affiliations:** 1 Lee County Mosquito Control District, Lehigh Acres, FL USA; 2 Insect Pest Control Subprogramme, Joint FAO/IAEA Centre of Nuclear Techniques in Food and Agriculture, Department of Nuclear Sciences and Applications, International Atomic Energy Agency, 1400 Vienna, Austria; 3 Department of Entomology and Nematology, University of Florida, Gainesville, FL USA; 4 Division of Vector-Borne Diseases, Centers for Disease Control and Prevention, Fort Collins, CO USA

**Keywords:** Sterile insect technique, Mosquito control district, *Aedes aegypti*, Public health

## Abstract

**Background:**

Lee County Mosquito Control District (LCMCD) is an independent taxing district that works to protect human health and improve quality of life in Lee County, Florida, USA. With local dengue transmission in southern Florida, LCMCD prioritized the control of *Aedes aegypti.* Due to the cryptic larval habitats of *Ae. aegypti* and insecticide resistance, effective control using conventional methods is difficult. Thus, the sterile insect technique (SIT) program, using X-ray irradiated male mosquitoes, was created to target *Ae. aegypti.* The goal of this program was to suppress *Ae. aegypti* through establishing a robust SIT program and performing a pilot study in the field to assess the impacts of SIT releases.

**Main text:**

The SIT program at LCMCD released sterile male *Ae. aegypti* from 2020 to 2022 in Captiva Island, Florida. The SIT program works within a larger Integrated Mosquito Management (IMM) framework and is not a standalone tool. The SIT program consists of nine employees, one of which is dedicated to quality assurance. Quality assurance assessments are performed routinely and periodically. Due to widespread destruction throughout Captiva and Sanibel Islands from Hurricane Ian in September 2022, the SIT pilot in Captiva Island was concluded and moved to Fort Myers, Florida. During the pilot study on Captiva Island, various lessons were learned and this knowledge has been applied to efforts in Fort Myers.

**Conclusions:**

LCMCD has established a successful SIT program to suppress populations of *Ae. aegypti*. Through connections with the International Atomic Energy Agency (IAEA) and the University of Florida, LCMCD received guidance from experts in the field to help ensure the program’s success. Stable funding through taxes levied specifically for mosquito control provided essential consistency, allowing the program to grow and evolve. Consistent trapping routines provided immense amounts of entomological data. Thoughtful and intentional community engagement was essential in ensuring acceptance of the SIT program in Lee County. Following the phased conditional approach suggested by IAEA, LCMCD has built an effective and resilient SIT program. The integration of the SIT as a tool of an area-wide mosquito control program is a feature that distinguishes LCMCD’s SIT program from others.

## Background

Lee County Mosquito Control District (LCMCD) is located in Southwest Florida, USA and is the largest single county mosquito control district in the United States. LCMCD was established as an independent taxing district in 1958 and is tasked with improving quality of life and protecting the public health of the residents of Lee County [1]. As a special taxing district in the state of Florida, a specific tax is levied from the property owners of Lee County, FL [1]. A millage rate is set by LCMCD’s board of commissioners, and taxes raised go directly to LCMCD. This provides stable funding that is specifically dedicated to mosquito control. Historically, mosquito suppression efforts have focused on highly pestiferous and abundant species such as *Aedes taeniorhynchus* (Wiedemann), which have necessitated large scale area-wide mosquito management efforts. However, efforts have evolved to include control techniques specifically targeting the disease vector *Aedes aegypti* (Linnaeus) [2].

*Aedes aegypti* is an anthropophilic mosquito that occupies natural and human-made containers, making it a species that thrives in urban and suburban habitats. Although LCMCD can effectively suppress mosquito populations using traditional mosquito control techniques, such as larviciding and adulticiding, these are largely inefficient against *Ae. aegypti* [3]. While source reduction is ultimately the best way to reduce populations of *Ae. aegypt*i, this is not realistic on a broad scale [4, 5]. *Aedes aegypti* has been a long-term pest throughout much of Lee County, but given the local transmission of dengue in the Florida Keys in 2010 and the spread of chikungunya and Zika in Florida, there was growing concern for the dangers posed by these mosquitoes [6, 7]. Additionally, widespread insecticide resistance to pyrethroids was documented in populations of *Ae. aegypti*, including those in Lee County [8]. LCMCD began pursuing the sterile insect technique (SIT) in 2016 in an effort to reduce the threat to public health posed by *Ae. aegypti* [9].

The SIT is a method of pest control using area-wide and massive releases of sterile male insects to reduce pest populations [10]. The SIT has a long history in Florida as this is where the first operational use of this technology occurred in attempts to eradicate the New World screwworm, *Cochliomyia hominivorax* (Coquerel), from the United States [11]. This technology has often been used to control agricultural pests, yet there are various programs currently evaluating the use of the SIT to suppress populations of *Ae. aegypti* in different countries [12] Due to the challenges faced through controlling *Ae. aegypti* with conventional methods and concerns for possible disease transmission in the future, LCMCD sought to implement a SIT through conducting a pilot study in the field and the establishment of a program.

## Main text

### Creation of the SIT program at Lee County Mosquito Control District

In April 2017, LCMCD met with representatives from the Joint Program Food and Agriculture Organization (FAO)/International Atomic Energy Agency (IAEA) Insect Pest Control Section in Vienna, Austria to discuss the implementation of the SIT for *Ae. aegypti* in Lee County. This technology transfer meeting provided the foundational information needed to begin a pilot project. Using a phased conditional approach to beginning a SIT program, LCMCD received guidance on essential components of SIT for *Aedes*, such as community engagement, mosquito irradiation, entomological baseline collection, mosquito mass rearing, and quality assurance methods, among other topics [13]. LCMCD was the ideal option to create a mosquito SIT program in Lee County as it would be a part of a comprehensive mosquito control operation. Following best practices for Integrated Mosquito Management (IMM), LCMCD would be able to control mosquito population with traditional techniques while also integrating this novel intervention. Effective mission planning between chemical mosquito suppression methods and sterile male releases would allow for minimal negative impacts to released sterile males. By selecting which mosquitocidal product would be applied and when, the LCMCD SIT program would be best able to employ the SIT as a part of an area wide-IMM approach. Based on this meeting, it was determined that Captiva Island would serve as the optimal pilot site for implementation of a SIT program to control *Ae. aegypti*. As a barrier island that was accessible by automobile but removed from the mainland, Captiva Island provided an ideal location that would have very limited immigration of this mosquito species [14]. Additionally, Captiva Island has a known population of *Ae. aegypti* with no known overlap of *Aedes albopictus* (Skuse), reducing concerns over potential population replacement from another similar container-inhabiting mosquito. A neighborhood at the northwesternmost end of Sanibel Island was selected as the non-intervention area to monitor alongside Captiva Island due to its proximity and similar habitat to Captiva Island.

**Fig. 1:**
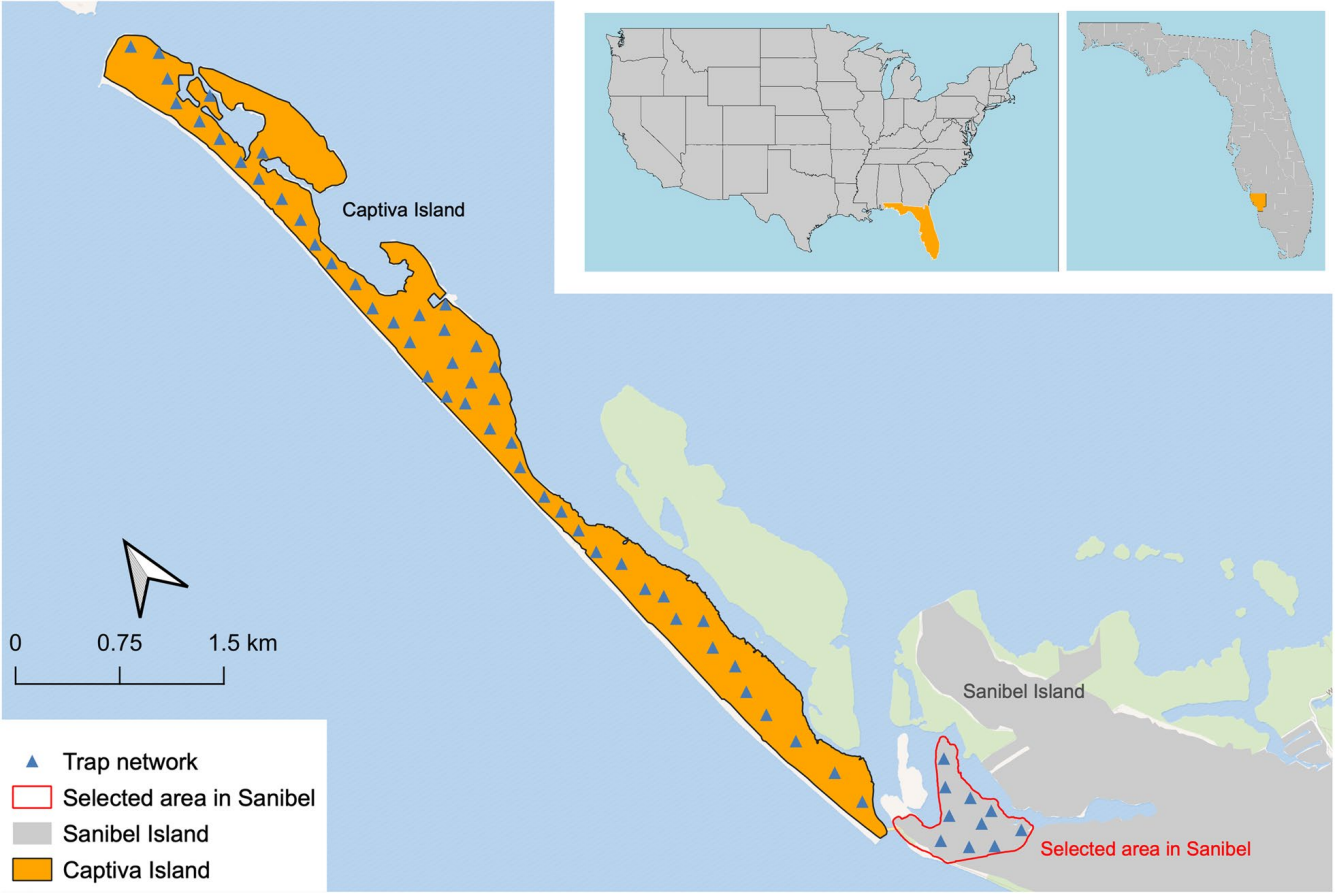
Map of Lee County Mosquito Control District’s sterile insect technique pilot location, Captiva Island, and non-intervention location, Sanibel Island, with trap locations (OpenStreetMap.org) [15]

Collection of the entomological baseline began in June 2017 and was comprised of adult surveillance with BG Sentinel 2 (Biogents AG, Regensburg, Germany) traps paired with oviposition cups for egg surveillance (Fig. 1) [15]. Through oviposition cup egg collections, LCMCD was able to establish a colony of *Ae. aegypti* for mosquito mass rearing. With this strain, LCMCD used a Rad Source 2400 V (Rad Source Technologies, Buford, GA, USA) and established dose response curves in response to X-ray irradiation, defining 42 Gy as the required dose to achieve male sterility without negatively impacting fitness. Releases of sterile male *Ae. aegypti* began on June 10, 2020, and continued until September 23, 2022.

The IAEA was instrumental in establishing a SIT program at LCMCD. From the initial planning meeting to eventually hosting a visiting scientific expert, IAEA has played a vital role in many aspects of the SIT program. LCMCD has also received extensive support from Dr. Daniel A. Hahn of the Entomology and Nematology Department, University of Florida. Dr. Hahn aided in fine-tuning various components, especially in dosimetry and dose–response. LCMCD also received additional financial support through a Centers for Disease Control and Prevention-Florida Department of Health Hurricane Cooperative-Agreement grant (CFDA No. 93.323) from 2019 to 2020.

### Logistics of the SIT program at Lee County Mosquito Control District

#### Personnel

The SIT department has a dedicated staff of nine employees with responsibilities that are strictly related to their assigned tasks within the SIT program. Having committed personnel is critical to overall quality and consistency in mosquito production, sterile male releases, and field surveillance. The department is overseen by a manager who plans and implements strategies for surveillance and releases. Three assistants perform the field work of setting and collecting traps, releasing sterile male mosquitoes, and assisting with insectary cleaning tasks. Three technicians have the responsibilities of mass rearing *Ae. aegypti*, maintaining colonies, preparing and irradiating male mosquitoes, and assisting with quality control measurements. One supervisor directs the daily activities of the assistants and technicians, ensuring that trapping occurs consistently, and sterile males are prepared for releases. A quality control specialist performs various assessments to measure and track the health and fitness of mosquitoes reared for both colony maintenance and sterile male production, ensuring that the female contamination threshold of 0.2% is not exceeded, and providing guidance for possible points of improvement in the rearing process. 

#### Quality assurance

Every batch of mosquitoes is assessed for a variety of indicators of quality and fitness. Routine assessments allow production to be tracked over time, aiding in the detection of any negative trends. These routine assessments begin with data collection of the hatch rate of eggs used to start each batch. At the pupal stage, after the separation of males from females, pupae are weighed, and samples are visually inspected to confirm that the female contamination threshold of 0.2% has not been exceeded. The pupal weights are important measurements that allow for better estimations of production, male protandry, and can also help determine larval rearing problems. Pupal weights are considered along with the other routine assessments to evaluate the quality of the batch and are tracked over time to monitor colony health. Dosimeters are used with every X-ray irradiation cycle to verify that the appropriate dose has been achieved. After pupal irradiation, a sample of male pupae is retained and compared with non-irradiated males in terms of emergence. Emergent adults are then monitored for survival under stress, where they are given only water and mortality is recorded daily, resulting in Kaplan–Meier (K-M) survival curves. Males prepared for field releases are allowed to emerge in modified paint buckets. After emergence is complete, pupal mortality is assessed by inspecting the drained water from the release buckets. After field releases are complete, buckets are returned to the laboratory and mortality is quantified for any remaining adult mosquitoes to help better estimate the number of released mosquitoes in the field.

Several periodic assessments are also conducted annually to check the fitness of the laboratory colony. Sex ratio examination indicates how many males compared to females are produced allowing for more accurate production estimations. While immature development time is indirectly recorded through the production of sterile male batches, it is also a dedicated periodic assessment to specifically monitor the rate of larval growth and density, the feeding regime, and the food source which aids in more accurate planning for male production. To assess the longevity of the males produced, an extended male survival experiment (offering a sucrose solution) is conducted with irradiated and non-irradiated mosquitoes, evaluating daily mortality, and adjusting a K-M curve. Wing length is used to compare the adult size of the laboratory colony to the wild population at the release site. Mating competitiveness is assessed through tests conducted annually or whenever significant changes are made to rearing or irradiation procedures, following the method proposed by Pagendam [16]. A cage with a 1:1:1 ratio of irradiated males, unirradiated males, and virgin females is allowed to mate for a 24-h period. After which, the males are removed and the females are blood-fed. Each female is then transferred to an individual oviposition chamber. The eggs are subsequently flooded, and the Fried index is calculated to provide robust and reliable data for comparative analysis. Eggs are aliquoted based on weight; therefore, an accurate egg weight is essential to correctly estimate the number of eggs required to produce appropriate amounts of mosquitoes. As egg weight is a very stable measurement, this assessment can be performed annually or biennially. Mark-release-recapture studies (MRR) are very labor intensive but provide critical information necessary for sterile male release planning, such as population size, sterile male dispersal, and longevity of released males [14]. Conducting MRRs on a quarterly basis allowed for the examination of seasonal impacts on sterile male releases and population sizes. Overall, sterile males had an average life expectancy of ~ 2.46 days with a mean distance traveled of 201.7 m. After the initial foundational information from MRRs is obtained, MRR frequency can be reduced to an as-needed basis. Calibration of the X-ray irradiator is performed annually but is also required after significant machine maintenance or when new dosimetry films are introduced.

#### SIT releases and evaluation

Due to impacts from COVID-19, LCMCD was forced to delay releases of sterile males on Captiva until June 2020, missing the optimal window of seasonally low *Ae. aegypti* populations. In order to help reduce the initial population, LCMCD applied a treatment of area-wide larvicide followed three days by adulticide. Releases began on June 10, 2020 and scaled up over time, growing from approximately 100,000 to 400,000 sterile males released per week. In general, males were released at a rate of 2200 sterile males per hectare, although this varied based on actual production outputs and increasing releases based on areas of high mosquito activity. Although there were lower *Ae. aegypti* populations during winter, there was still known population activity throughout the year [15]. As a response, LCMCD chose not to scale back releases seasonally, instead expanding the release area when possible due to alternating release densities due to reductions in mosquito hotspots and sterile male production increases. Since the SIT program was considered a part of the overall IMM approach, chemical interventions of larvicides and adulticides targeting other mosquitoes continued as needed in both Sanibel and Captiva. However, special planning was used to mitigate insecticide exposure to released males.

The network of traps and oviposition cups that provided the data for the entomological baseline are also used to evaluate impacts from sterile male releases. Eggs collected in oviposition cups were returned to the lab where they were counted and assessed as appearing viable, non-viable, or hatched in the field. Eggs that appeared viable were hatched and reared to adults to determine hatch rate, species, and sex. The percentage of eggs that hatched was calculated by adding the eggs hatched in the field to those hatched in the lab, dividing this amount by the total number of eggs collected, and multiplying this amount by 100.

All sterile male *Ae. aegypti* that were released were marked with fluorescent powder (DayGlo Color Corp., Cleveland, OH, USA). Releasing marked males allowed the opportunity to distinguish between wild vs. released *Ae. aegypti*. The ability to differentiate between marked and unmarked mosquitoes yielded increased accuracy for population measurements. Another advantage gained by marking all released males was through feedback on the longevity of released males by alternating pigment used to mark males on a weekly basis.

#### Community engagement and acceptance of the SIT in Lee County

Captiva Island presented a unique situation for how outreach is normally conducted by LCMCD. Traditionally, residents would be the primary focus of education efforts [17]. On Captiva Island, however, there are very few permanent residents with the vast majority of homes and condominiums functioning as vacation homes or rental properties. Instead of the typical residential approach, we met with the Captiva Island Chamber of Commerce to familiarize business leaders with the project. We then participated in a meeting with biologists from Captiva and Sanibel Islands to inform them about our plans to suppress *Ae. aegypti* with the SIT. Town hall meetings were held to educate citizens, address any concerns, and answer questions from the public. LCMCD employs teachers with the Lee County School District who educate children in area schools about various aspects of mosquito control, during which they began to introduce the concept of SIT in their lesson plans. In addition to in-person engagement, we also worked extensively with local media. We invited local news crews at various times to join us for mosquito releases, MRR studies, and to observe our mosquito rearing at the district. Our efforts were also highlighted on the front page of the local newspaper [18]. Remote interviews via phone and virtual meetings were conducted as well, providing an even broader outreach. Once our releases began, we started fielding questions from an interested public. Each release served as an educational opportunity and visitors to the island would frequently ask what we were doing. This direct public engagement was outstandingly positive and left many vacationers wondering about the mosquito control efforts in their own areas and if they could have a similar SIT program. There were no additional notification efforts regarding the conclusion of our pilot project on Captiva Island. Public acceptance of the SIT for mosquito control was extremely high throughout our release period. Although it would have been beneficial to perform pre and post intervention surveys to gauge individual acceptance of the intervention, acceptance was instead inferred through the overall lack of complaints. While we received a few requests to move trapping locations, pushback against the release of sterile males was virtually nonexistent. The public embraced a non-chemical approach to control a dangerous mosquito. By explaining that sterilization of males was induced via X-ray and relating it to what they would experience in medical procedures, they felt that this was a safe procedure. Clarification between the SIT and genetic modification was also important to reduce hesitancy.

#### Considerations for a SIT program to control West Nile virus vectors

While there is interest in adapting the SIT for West Nile virus vectors, such as *Culex nigripalpus* Theobald and *Culex quinquefasciatus* Say, this presents increased challenges compared to *Aedes* vectors. *Aedes* eggs can dry and be stored for extended periods of time, whereas *Culex* eggs cannot survive if dried out. This results in the inability to build a storable egg bank with production amplification difficult at best. Furthermore, since the SIT depends on an overflooding ratio for success, releases would not be appropriate for species that are too abundant in the wild, as is the case for *Cx. nigripalpus* and *Cx. quinquefasciatus* in Lee County [1, 5]. The SIT for *Culex* suppression is currently not a viable option in Lee County given the available technology and program constraints.

#### Post Hurricane Ian

On September 28, 2022, Hurricane Ian made landfall which effectively concluded the pilot project on Captiva Island. Hurricane Ian was an exceptionally destructive tropical cyclone that made landfall in Lee County on Cayo Costa Island as a high-end category 4 storm [19, 20]. This storm utterly devastated Sanibel and Captiva Islands. The Sanibel Causeway, the only route on or off the islands, was damaged in five sections, with portions of the road washed away. Thus, access to the islands by automobile was eliminated for months until enough stabilizing repairs were completed. Beyond accessing the pilot area, the habitat had been radically altered, with salt water from the storm surge intruding into nearly all larval sites, vegetation was substantially reduced, and homes had various levels of damage. Due to these factors, it was necessary to conclude the pilot releases on Captiva Island and relocate to a new area. Although the general public was not notified regarding the conclusion of the pilot program, our traditional mosquito control operations remained ongoing. This, coupled with slow recovery on the islands, resulted in no impact on public relations due to the cessation of release activities. The results of this project have been communicated to the public through presentations with various local community organizations.

The selection of a new area for SIT intervention began with candidate communities that had a history of *Ae. aegypti* presence. From these locations emerged several options that also had favorable environmental features that would reduce immigration of mosquitoes into the release area. After several trapping events, a new focus area was selected in Fort Myers. Adult and egg surveillance began in December 2022 at the same schedule and trap density as Captiva and Sanibel Islands to establish the entomological baseline in the new area. Several MRR studies have also been conducted to estimate the population of *Ae. aegypti* in the new location and to investigate if there were differences in male dispersal. Sterile male releases began in February 2024 and are ongoing. Initial feedback through surveillance has shown impacts to the population of *Ae. aegypti*, with similar trends as were observed in the pilot study on Captiva.

#### Lessons learned

Having gained experience in all facets of a comprehensive SIT program, there have been a myriad of opportunities to learn from and improve the process. Some of the key lessons learned have been summarized in Table 1. Reference values are important benchmarks that are critical to the quality control process. As was detailed in the Quality Assurance (QA) section, certain measurements should be made periodically in order to monitor the long-term health of the colony. While these eventually became a part of the QA procedures, it would have been useful to incorporate them from the beginning. Although LCMCD has an extremely robust colony that was developed from Captiva Island, it would have been useful to obtain the initial QA measurements so that colonization effects, both positive and negative, could be better understood.

**Table 1:** Summary of Lee County Mosquito Control District’s most significant lessons learned

Lessons learned
Increased production rate with larval rearing system and pupal separation system
Optimized workflow with moving from pupal irradiation to adult irradiation
Reduced burden of data entry and analysis with the development of an in-house database that was curated specifically for our SIT program
References values from the beginning are essential for quality assurance
Product inconsistencies in larval diet and blood can lead to unexpected colony complications

Additionally, it would have been better to consistently assess the eggs collected from the field. Originally, eggs were assessed only as viable, nonviable, or hatched. Eventually a subsample was assessed for the hatch rate in the lab. As egg collections were not consistent throughout the sites, it was ultimately most beneficial to assess the hatch rate on all eggs collected from the field. This gave the clearest picture of the actual viability of the eggs, along with the detection of other species that occasionally used oviposition cups. While full assessment of the eggs is more labor intensive, the results are ultimately more useful.

Fortunately, there are advances in equipment necessary to rear larvae and separate mosquito pupae. Initially, LCMCD began rearing *Ae. aegypti* in large 6 L pans that were used for previous unrelated mosquito rearing needs at the district. The pans were cumbersome and inefficient with respect to pupal removal, having a need to be drained individually and slowly. By switching to a larval rearing rack system that was designed to mass-rear mosquitoes (Guangzhou Wolbaki Biotech Co., Ltd, Guangzhou, China), the process of draining pupae was expedited. Additionally, the rack systems contain closely spaced larval rearing pans which allow for rearing greater quantities of mosquitoes in a smaller area. LCMCD currently has six larval rearing rack systems. This greatly expanded the mass-rearing capabilities of our SIT program. Separation of male and female pupae presented the next bottleneck. A Fay-Morlan glass plate separator was initially used to separate male pupae from female pupae [21]. This was a time-consuming process that required a skilled technician to achieve low female contamination rates. Even with experience, contamination rates were inconsistent between staff members and the volume that could be separated was too low for mass production. The acquisition of two automatic pupae sex separators (Guangzhou Wolbaki Biotech Co., Ltd, Guangzhou, China) proved essential for scaling up production, reducing human error, and speeding up the process [22, 23].

In time the amount of data that was being collected through all the various points of the SIT program became overwhelming. Without having a coordinated database, the data were difficult to manage. LCMCD has a computer programmer on staff who was able to construct a database capable of handling the data generated from both field and lab activities. The database was built with an SQL server (Version 2014). A program was built with C# (desktop) and was used to interact with the database. It proved extremely beneficial to have a database specifically designed for the needs of the program, especially as it included proofing to ensure accurate data entry. Some of the primary uses were recording results from mosquito trapping, egg surveillance, and the information associated with sterile male releases, among other data. Many of these could be directly exported to Microsoft Excel. The use of a dedicated database has helped to protect the data, reduce data processing time, and has assisted with further analysis. Up to date data analysis was essential for informed decision making.

**Fig. 2:**
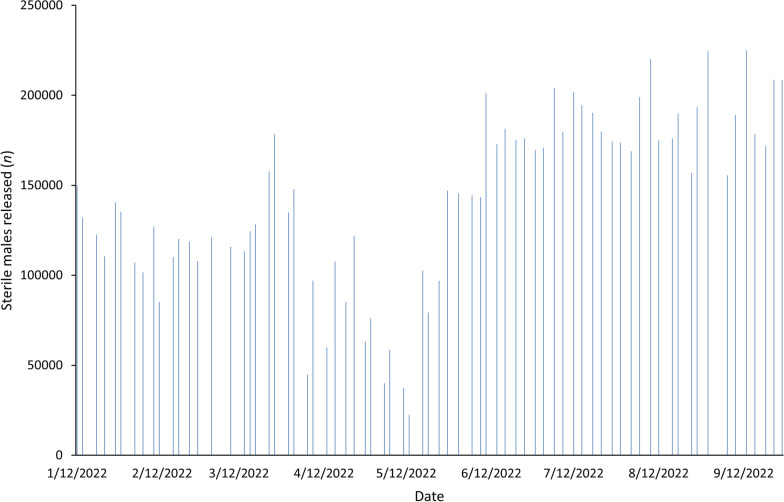
Sterile male *Ae. aegypti* production levels for 2022. Male production began declining in April 2022 due to inconsistencies with the frozen pork blood that had previously been used for colony maintenance. Male production was stabilized once the blood source was switched to defibrinated sheep blood

Larval diets are extremely variable, with institutions often creating formulations based on locally available ingredients [24]. To reduce time spent by staff in mixing larval diet ingredients and to reduce human error or inconsistencies in combining components in other larval diets, Purina® Aquamax® Fry Powder (Purina Mills, LLC, St. Louis, MO, USA) was used for larval rearing. Unfortunately, in time it became clear that there were differences in product consistency between production facilities. This was coupled with supply chain issues and rising costs of the product. Also, the odor generated by using this product for mass rearing became overpowering. By converting to a different formulated product, Zeigler® Tropical Pond Meal (Zeigler Bros., Inc., Gardners, PA, USA), the advantage of reduced effort by staff and less potential for human error were maximized. This product was consumed entirely by the larvae, with less waste and debris generated which ultimately reduced the odor in the larval rearing room. Similarly, the blood feeding of adult mosquitoes was complicated by product consistency issues. The SIT program was able to support colonies of mosquitoes using food-grade frozen pork blood for several years [25]. In April 2022, it became clear that a significant change in the blood had been made which led to a decline in colony egg production. The alterations were so substantial that the frozen blood was no longer a viable option, requiring an abrupt shift to defibrinated sheep blood in mid-May 2022 (HemoStat Laboratories, Dixon, IL, USA). The egg production values were directly reflected in the sterile males produced for the field and resulted in reduced amounts released from April to May 2024, until blood feeding was stabilized with defibrinated sheep blood (Fig. 2).

Synchronization of pupation is important to efficiently separate male and female pupae and maintain the correct window required for pupal irradiation [26, 27]. While rearing larvae on a smaller scale had not presented challenges with synchronization, increasing to mass rearing amplified any inconsistencies in development. Due to satisfactory egg hatch rates, it was determined that the greatest factor in the cause of the asynchrony in this situation was differences in larval feeding times. Variation in the time when the larvae were fed throughout their development (for example morning one day and afternoon the following day) resulted in a more asynchronous batch, reducing the males collected and increasing waste. By adhering to a schedule with designated feeding times, the overall synchrony and productivity was much improved.

#### Coordinated mosquito control efforts

Incorporating the SIT into a comprehensive mosquito control operation is an important contributor to a program’s success. In combination with source reduction (when possible), community education, and chemical control methods, the SIT was integrated into the overall IMM program at LCMCD. Any larviciding or adulticiding activities that had the potential to impact *Ae. aegypti* in the pilot area, whether *Ae. aegypti* was the intended treatment target, was communicated and recorded to document potential changes in population. Prior to releasing sterile males on Captiva Island, a low volume area-wide larvicide mission and a separate adulticide mission were conducted to help suppress existing populations of *Ae. aegypti*. These initial conventional control measures, such as larviciding and adulticiding are important to ensure successful SIT releases, as the SIT is more effective during low population density periods [28]. The incorporation of multiple control measures also can be beneficial in enhancing cost effectiveness [12]. Once releases began, careful coordination between the SIT program and the mosquito operations department helped prevent sterile released males from being treated with adulticide. No specific chemicals or traditional mosquito control interventions were avoided as there is a limited variety of chemicals available for mosquito suppression and we needed to rotate the different products to reduce potential for insecticide resistance. Instead, the release schedule could be altered if it was required to accommodate a pressing mosquito treatment. By working alongside other mosquito control efforts, the SIT program was able to be much more effective than as a standalone intervention.

#### Future work

LCMCD is actively continuing this program in Fort Myers, Florida, with plans to expand production and releases. Using the entomological baseline obtained through surveillance in the previous year, LCMCD began sterile male releases in late February 2024. With the re-establishment of consistent mass rearing there is the need and opportunity to scale up production. LCMCD intends to expand releases to one million sterile male *Ae. aegypti* per week. This increase in production will be facilitated by the conversion from pupal irradiation to adult irradiation. To achieve the proper dose for pupae that induces sterility without negative health impacts, a tight window of pupal ages must be irradiated [29]. Adult irradiation has the combined benefit of reduced sensitivity of adults along with a greater time window to effectively sterilize adults than with pupae, resulting in a more flexible workflow [30]. While a similar number of steps are involved in both pupal and adult irradiation, the duration from processing pupae to release-ready males is quite different with pupal irradiation taking up to 86.67 h and adult irradiation taking up to 60.42 h (Table 2). Progress has already been made to research the basic parameters necessary for this change with full conversion expected in 2025 [31]. In addition, a cost effectiveness assessment in partnership with IAEA was conducted. As *Ae. aegypti* continues to pose a significant health threat and with limited effective tools at our disposal, the SIT is a part of the overall strategy of disease prevention through suppressing this resistant and highly adaptable species.

**Table 2:** Steps involved with pupal and adult irradiation with associated times

Pupal irradiation	
Task	Time duration
Individual pupal weights	30−40 min
Return to holding container for overnight	Max. 16 h
Drying and aliquoting	Max. 2 h
Loading canisters, irradiation, removing pupae	10 min, repeated 3 times
Place in emergence containers with sugar	30 min
Emergence	~ 36−48 h
Draining	2 h
Dry overnight	Max. 16 h
Marking	1 h
Ready for release	Total time: up to 86.67 h

Adult irradiation	
Task	Time duration
Individual pupal weights	30−40 min
Drying and aliquoting	Max. 2 h
Place in emergence containers with sugar	30 min
Emergence	~ 36−48 h
Draining	2 h
Dry containers	30–60 min
Cold knockdown	75 min, repeated 2−3 times
Packaging	30 min, repeated 2−3 times
Loading canisters, irradiation, removing adults	5 min, repeated 2−3 times
Marking and return to container	15 min, repeated 2−3 times
Ready for release	Total time: up to 60.42 h

## Conclusions

The SIT is an effective tool that should be considered to suppress certain mosquito populations, especially those with limited flight range and where overflooding ratios can be achieved. LCMCD has established a successful SIT program to suppress populations of *Ae. aegypti* using X-rays. By establishing connections with the IAEA and the University of Florida, LCMCD was able to obtain continued guidance from experts in the field. The stable funding source for LCMCD through taxes levied specifically for mosquito control provided essential consistency, allowing the program to grow and evolve. Adhering to an intensive and consistent trapping schedule provided a wealth of information for the entomological baseline. Prioritizing quality assurance was an important factor that was essential to produce consistent amounts of males that were fit and competitive in the field. A vital part of an effective SIT program, LCMCD engaged with the public in a variety of ways to disseminate information about this new suppression effort by the district. Using in-person meetings, print, and television, a broad audience was reached and educated about the benefits of using the SIT in an integrated intervention for *Ae. aegypti* suppression. Utilizing consistent surveillance of mosquitoes which included a non-intervention area, LCMCD was able to observe impacts and declines in the population of *Ae. aegypti* in the release area. Although other mosquito species, especially vectors of West Nile virus, are of interest as targets of the SIT, at this time LCMCD remains focused on *Ae. aegypti*. 

Unfortunately, Hurricane Ian took a devastating toll on Sanibel and Captiva Islands in September 2022. It was necessary to conclude SIT-related operations on Captiva Island due to challenges in accessibility, overall community impacts from the storm, and significant habitat changes. While this was a setback to the program, it allowed the SIT to be applied to a new area. Using the knowledge and experience gained from the pilot project on Captiva Island, LCMCD is focused on an area in Fort Myers with a known historical *Ae. aegypti* presence. LCMCD is actively continuing this program in Fort Myers with plans to expand production and releases. As *Ae. aegypti* continues to pose a significant health threat and with limited effective tools at our disposal, the SIT is a part of the overall strategy of disease prevention through suppressing this difficult to control species.

Following the phased conditional approach recommended by IAEA, LCMCD has built an effective and resilient SIT program. The integration of the SIT as a tool of an area-wide mosquito program is a feature that distinguishes LCMCD’s SIT program from others. Being part of the agency that directly suppresses mosquitoes throughout the county gives the SIT program an edge to coordinate sterile male releases with mosquito treatments, adding a valuable tool to our IMM program. 

## Data Availability

Not applicable.

## Abbreviations

LCMCD: Lee County Mosquito Control District
SIT: Sterile insect technique
IMM: Integrated mosquito management
IAEA: International Atomic Energy Agency
MRR: Mark release recapture
FAO: Joint Program Food and Agriculture Organization
K-M: Kaplan–Meier
